# Supplementary material for: A ddRADseq Survey of the Genetic Diversity of Rye (Secale cereale L.) Landraces from the Western Alps Reveals the Progressive Reduction of the Local Gene Pool
Source: Plants (Basel). 2021 Nov 9;10(11):2415. doi: 10.3390/plants10112415 (PMC8624659; doi:10.3390/plants10112415)
Supplement: Supplementary file 1 [file plants-10-02415-s001.zip › plants-1402241-supplementary.pdf]

plants

MDPI

A ddRADseq survey of the genetic diversity of rye (Secale cereale L.) landraces from the Western Alps reveals the progressive reduction of the local gene pool.

Supplementary materials

Martino Adamo, Massimo Blandino, Luca Capo, Simone R. Enri, Anna Fusconi, Michele Lonati, Marco Mucciarelli

1. Supplementary Tables

Table S1. Evanno method results.

K	Reps	Mean LnP(K)	Stdev LnP(K)	Ln'(K)	|Ln''(K)|	Delta K
1	10	-1941440.46	380.510076	NA	NA	NA
2	10	-128345.000	2.339	15465.300	4423.940	81.389
3	10	-124792.860	5.452	3552.140	1714.040	314.373
4	10	-122954.760	271.332	1838.100	97.720	0.360
5	10	-121018.940	407.545	1935.820	118.640	0.291
6	10	-119201.760	43.523	1817.180	1107.370	25.444
7	10	-118491.950	1236.175	709.810	562.160	0.455
8	10	-117219.980	323.733	1271.970	600.870	1.856
9	10	-116548.880	251.563	671.100	131.370	0.522
10	10	-115746.410	79.609	802.470	131.623	1.653
11	8	-115075.563	106.352	670.848	NA	NA

2. Supplementary Figures

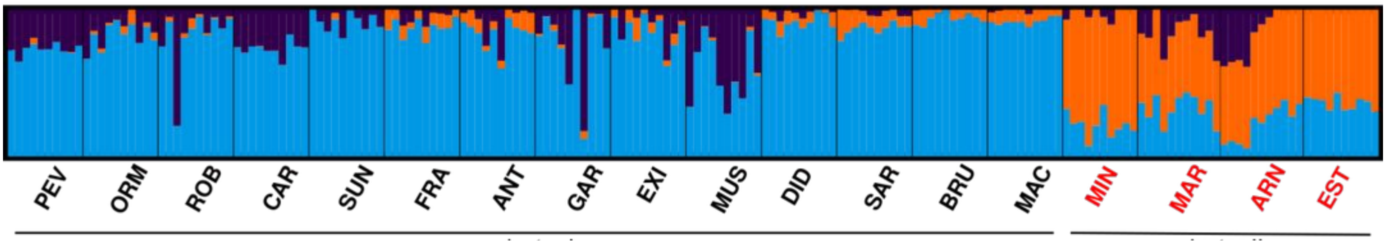

Figure S1. Structure output for K = 3. Interpretation of the STRUCTURE results with Evanno method (Evanno et al., 2005) for the most likely K = 3. Rye samples are separated in three main genetic clusters. Accessions are statistically assigned to two main groups; cluster I (black IDs) and cluster II (red IDs).

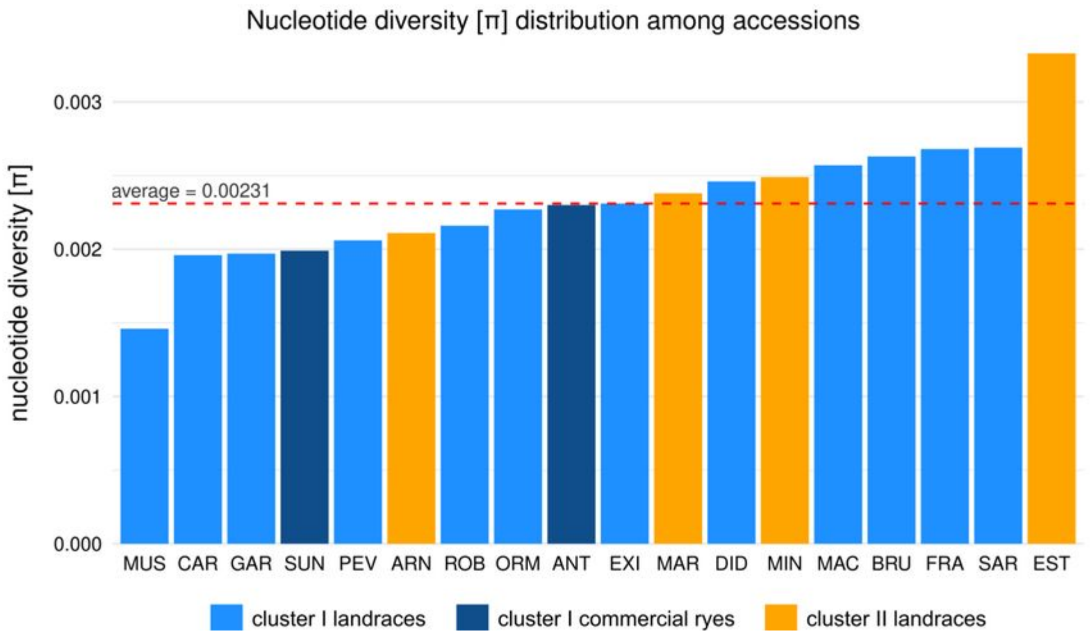

Figure S2. Nucleotide diversity (π) distribution among rye accessions. Populations are ordered by the ascending value of π. Bright and dark blue bars are for accessions belonging to cluster I, orange bars are for accessions belonging to cluster II. Dark blue differentiate commercial rye accessions from landraces in cluster I. Red dashed line corresponds to the average π.

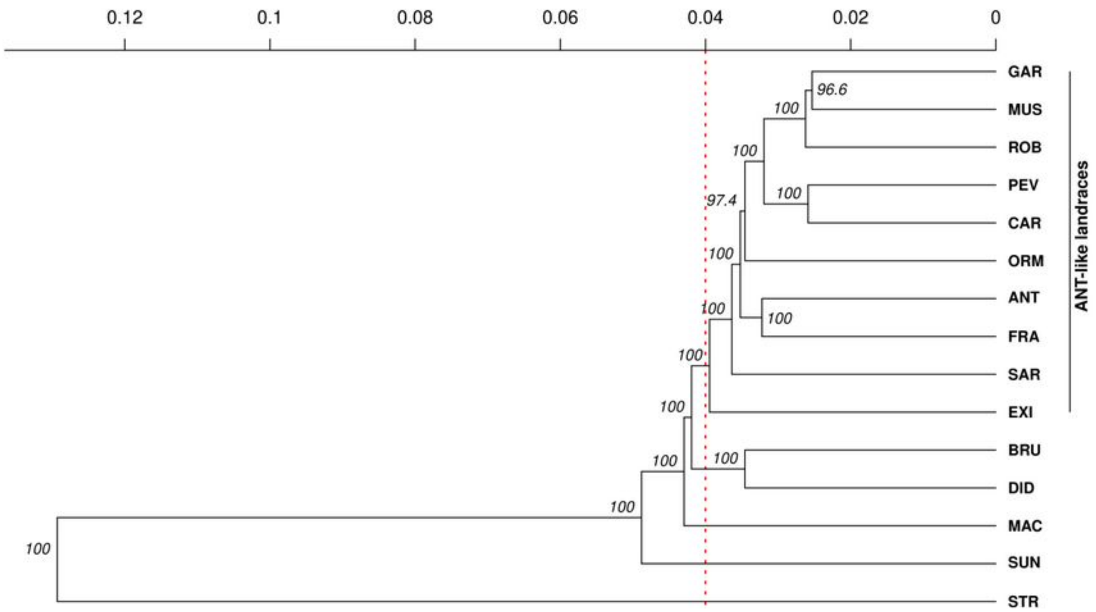

Figure S3. Neighbour joining tree based on Prevosti's genetic distances between accessions from cluster I. Populations are those from the cluster I in Structure, with the addition of *S. strictum* as outgroup. We selected 0.04 as a threshold (dashed red line) to inspect genetic relationships within the larger genetic cluster of rye landraces and the two modern varieties. Topology is nj-based and bootstrap values are reported only when higher than 75.
